# Whose trauma is it anyway? Creating more equitable mental healthcare in a system that harms

**DOI:** 10.1192/bjb.2024.103

**Published:** 2025-08

**Authors:** James Downs

**Affiliations:** Royal College of Psychiatrists, UK

**Keywords:** Trauma, iatrogenic harm, inclusion, complex trauma, lived experience

## Abstract

Past experiences of mental healthcare which have been perceived as harmful can present significant barriers to accessing treatment again. This article draws upon research and lived experience to consider the ways in which conceptualisations of ‘trauma-informed care’ may better incorporate the role of iatrogenic harm, thus providing more acceptable and equitable treatment for those who have previously found treatment to be harmful. A more restorative approach is offered, founded in shared responsibility and compassionate relationships, to help minimise harms and create a more healing system for patients and clinicians alike.

## Iatrogenic harm as a barrier to equitable care

All patients deserve timely and effective treatment. However, many groups of people do not have equal access to mental healthcare as it exists today. Even if care is obtained, it may not include and respond to the experiences and needs of diverse groups. For instance, those from ethnic and racial minorities are marginalised from mental health services by a range of additional barriers, including increased stigma, poverty and institutional discrimination.^1^ Within services, treatments that are offered are largely based on research conducted with narrow clinical samples that often underrepresent non-White participants or fail to report racial demographic information at all.^2^ There are many other groups who are underserved.

In my own experience, mental health services present an array of barriers. As an autistic person, the ways in which I am required to communicate with services are more likely to be designed to meet the needs and resources of service providers, rather than patients like me. For instance, having to opt in to a waiting list via an unscheduled telephone call increases the likelihood of me not making it onto the waiting list for treatment in the first place. Likewise, recognition of my difficulties as a male with an eating disorder has been lacking as a result of the highly gendered stereotypes about eating disorders from which I am somewhat excluded. Such restrictive conceptualisations of what illness may look like can leave those who do not fit feeling like their views and experiences are not understood and incorporated within treatment.^3^

Another barrier which I have perceived as equally important in my own experience, yet is often marginalised from discussion, is the role of iatrogenic harm (i.e. harm caused by healthcare and/or treatment). The lack of meaningful acknowledgement and responsiveness to past experiences of being harmed by mental healthcare has made it more difficult for me to engage in treatment again and has limited the ability of clinicians to help me. This has made treatment less effective and – at times – retraumatising.

Iatrogenic harm cannot be neatly segregated from other barriers to healthcare that have been mentioned. Being unfairly excluded and disadvantaged on the basis of differences that one cannot help can itself be damaging and can predispose individuals to specifically harmful forms of discrimination. These experiences may be interpreted by individuals as traumatic. Yet, when it comes to the ways in which I've perceived mental health services to conceptualise and respond to trauma, iatrogenic harm from mental healthcare itself has been conspicuous by its absence. This article seeks to create a space to consider the ways in which permitting this subject to have presence within clinical settings can facilitate the inclusion of patients like myself and benefit the provision of care that is more compassionate for all.

## Is trauma-informed care (TIC) really that inclusive?

In recent years, there has been growing recognition of the important role that developmental trauma and adverse childhood experiences can have in shaping health and illness throughout life.^4–6^ A substantial body of evidence now indicates how increased exposure to adverse childhood events (such as physical and emotional maltreatment, neglect, and deprivation) is associated with greater incidence of mental illness, substance misuse and poor health outcomes in adulthood.^7–9^ These have been theorised as having basis in the neurobiological changes resulting from trauma.^10–12^ In light of this evidence, service providers have sought to become more trauma-informed as organisations^13^ and to provide safe and inclusive support for individuals who have experienced trauma under the umbrella of TIC.^14^

TIC has been characterised in broad terms as offering safety, trustworthiness, choice, collaboration and empowerment to patients, with an emphasis on avoiding reactivating traumatic feelings and distress during treatment.^15^ However, there remains an ongoing need for greater consensus in how TIC is defined and unresolved questions about how responsiveness to past trauma may best be operationalised within service design, care pathways, staff training and treatment itself.^16^ For example, more needs to be understood about differential responses to adversity and why some individuals, groups and communities may suffer more lasting and severe impacts than others from the same kinds of traumatic events.^17,18^ This may relate to the role of resources, opportunities and strengths as much as the presence of adversity itself.^19–21^ There are also life experiences outside childhood which may be equally relevant, such as domestic violence, elder abuse and combat trauma.^22^

Alongside these, iatrogenic harm – and specifically trauma sustained in mental health services themselves^23^ – features in literature on TIC largely as something to be prevented by trauma-informed approaches, rather than a reason a trauma-informed approach may be needed in the first place. This is reflected in my own experience; I have met a profound resistance to recognising that it is even *possible* that I may have been harmed by treatment, despite perceiving the mental healthcare system as having harm built in.^24^ For example, I have been subjected to profound neglect and systematic denial of care when dangerously unwell. I lived with severe anorexia nervosa for more than 6 years before having any specialist treatment, often being excluded from services on the grounds of being too medically unstable or not motivated enough. At other times I haven't been visibly sick *enough*, incentivising me to become more unwell.

I've had relationships with mental healthcare professionals that have been coercive, been told stigmatising things about the kind of person I am, been diagnosed with a personality disorder by someone who had never spoken to me, experienced diagnostic overshadowing and late diagnosis of serious medical problems and neurodivergent conditions,^25^ been discriminated against on the basis of my differences, and experienced violation of my physical and sexual boundaries. I've had many things ‘done to’ me, but a lack of ‘care for’ or ‘being with’ me. Over many years of mixing with other patients, I have found that my experiences are not unique, yet they rarely have a place in discourse about what it is to be trauma-informed among the mental health professionals treating us.

The marginalisation of some kinds of trauma in favour of others prescribes a hierarchy of harms, where some traumas are considered more reasonable, likely and valid than others. In turn, people may feel invalidated in relation to their own experiences of harm (e.g. where it didn't occur in childhood), exacerbated by a lack of space in which to work therapeutically with their trauma. I have been in services which claim to be trauma informed but have only been interested in certain types of trauma, taking place at certain times and in certain places. Having such a limited and prescriptive understanding of what kinds of trauma services are willing to be informed about can result in clinicians being unable to comprehend and respond to more immediate harms that may be taking place in the here and now, proximal to – or even within – the clinical setting they operate in. If not rooted in a more expansive and inclusive conceptualisation of trauma, TIC may perpetuate and even generate fresh harms.

Distinction has been made between trauma-informed approaches to care and trauma-specific treatment which targets post-traumatic symptoms,^26^ but neither of these have seemed to accommodate my needs. I haven't wanted treatment for the trauma of past treatment when seeking help for an eating disorder, but nor have I found a trauma-informed approach to the kind of trauma I have experienced. Falling into this gap has been another form of harm.

## Stigmatising responses to trauma

Through my own experiences of adversity, I have learnt that it is more helpful for me to deal with reality as it is, rather than how I would like it to be: I can't erase my past trauma, so I have to accept it. Being willing to work with the responses that are evoked in me when seeking treatment is more effective than denying reactions that I cannot help having or expecting myself to behave as though I am someone who has not experienced iatrogenic harm – i.e. to be someone I am not. Psychotherapeutic change happens through the vehicle of relationship, and being someone you are not is hardly a good basis for authentic and meaningful relating.

As such, when seeking care in recent years, I have been open and honest from the outset about having had difficult experiences in the past. I have shared that this may make me appear ambivalent or distrusting at times, but that I want to name this so that it doesn't get in the way of a fresh chance for positive relationships. In more acute medical settings, I've disclosed at the first opportunity that I will find it difficult to stay in the hospital environment because of my past experiences, but that I want help to do so.

Rather than this being welcomed as valuable information for how to help me find care more acceptable, I have rarely been listened to by professionals, who seemed to resist the idea of iatrogenic harm in mental healthcare altogether. Instead, I've been met with defensiveness, which has felt divisive and undermined the opportunity for creating a trusting alliance. I've been attacked for bringing up past harm and characterised as wanting to punish professionals I have never met before for past injustices they were not involved in. Instead of my ability to be reflective being welcomed, I've been told that I am demonising and devaluing professionals, tarring them all with the same brush of my black and white thinking. I've been told that I demand perfect care, when raising concerns about the care on offer being fundamentally unsafe.

As well as making me feel blamed for the consequences of trauma that I live with, professionals have sometimes put considerable effort into digging for trauma that I do not have. I have been told that my illness is a result of my neglectful childhood, events that I cannot remember (including infantile sexual abuse) and that my mother is to be ‘forgiven’ for not knowing how to be a better parent. This would be a compassionate stance if it were true, but seeing as these explanations are false, this was just another form of blame in disguise. I imagine that anyone encouraged to look back at their childhood for difficulties, loss and pain when in a state of illness would be able to identify something that could be misidentified as a cause of present suffering. An overly narrow conceptualisation of trauma as largely situated in childhood also presents challenges of measurement, with formal questionnaires for childhood adversity having been challenged for their validity and reliability.^27,28^ Relying on retrospective identification is also problematic, as our interpretations of past events are heavily shaped by the present-day context from which we look back at them.^29^

The determination of my clinicians to situate harm elsewhere interests me, as I think it has often been less about finding accurate explanations and more about finding superficially acceptable, stereotypical ones which allow clinicians to avoid turning the lens back on themselves and the systems they operate within. This kind of fabrication may protect from the moral injury of working within services that are so under-resourced that they can often only hope to minimise harm.^30^ However, improving mental healthcare will only happen when we address rather than ignore the fundamental flaws in the status quo.

## From individual blame to shared responsibility

Blaming patients and their families for the occurrence and enduring impacts of trauma comes with an undoubtedly high cost and is an example of the tendency within mental healthcare to overly focus on ‘patient characteristics’ rather the qualities of the environments in which patients are situated (including healthcare). My view is that it is a mistake to attribute the fallout of broken systems to the individuals who are broken by such systems. Instead of over-privileging the role of the individual in healing the harms they have experienced, it is important for healthcare providers to acknowledge how trauma occurs in context and is often interpersonal. As such, trauma is a shared responsibility requiring collective action to redress.

The hyper-responsibilisation of individuals who are already facing the difficulty of being unwell can be extremely burdensome and in my own case has been the hallmark of engaging with healthcare at all. I have been expected to slot into services which do not seem to be designed for people with histories like mine. Care pathways are provided as though everyone will find them equally acceptable and be able to come afresh to a new mental health service (or return to one) as though they have never had a negative experience of mental healthcare before.

It would have benefited me hugely had the services I've encountered been designed with responsiveness to patients like myself in mind. It would help if they were willing to name iatrogenic harm as something that is possible, to allow space for it to exist and be recognised as important to work with in order to participate in treatment effectively. It would also be helpful if trauma-informed services were designed to equip staff with clinical skills to work with and even treat the consequences of traumatic experiences, including iatrogenic harm, rather than silo-ing therapies for trauma within specialist services. I hope we can stop patients having the experience that I have had within a community mental health team that claimed to offer TIC but said I was ‘too traumatised’ for the care on offer and so should be discharged to a waiting list for specialist trauma therapy more than 2 years long.

Preventing the exclusion of patients who have suffered iatrogenic trauma can be better achieved by including understanding of and responsiveness to iatrogenic harm within service design and models of care. Sweeney and Taggart^31^ provide an invaluable overview of the principles of TIC, which are rooted in a nuanced and broad conceptualisation of trauma, and identify a number of misconceptions and common pitfalls of trauma-informed approaches that would be beneficial for any care provider to consider, be that in service design or with respect to the skills of individual clinical practice.

There are alternatives to allowing iatrogenic trauma to overshadow the therapeutic process or remain in the shadows and limit the extent to which patients like me can be authentic in our relationships with clinicians. By facilitating disclosure, openness and a space to work with iatrogenic trauma, clinicians can help patients to work through this significant barrier with them. It may be restorative for patients who have experienced iatrogenic harm to have a different experience of being-in-relationship with a healthcare professional – one which does not deny their pasts. Clinicians may fear feeling attacked or as though they are being asked to take responsibility for harm they did not personally cause. Repair does not, however, require revenge or blame. Rather, clinicians need to assume responsibility as representatives of systems which have harmed and step into their power to provide a different, more positive relationship with that system.

## Whose trauma is it, anyway?

This article has been a way of creating a space to talk about the difficult and overlooked subject of iatrogenic trauma in mental healthcare. This has been especially important to me when, so often in my experience, the existence of my own iatrogenic trauma has been erased and even denied as a possibility. It may feel exposing, uncertain and overly complex to discuss the ideas that have been touched upon here, but I hope that this article demonstrates that it is possible and valuable to do so. Openness may help remove shame and challenge the need for stigmatising and blaming explanations for the difficulties that patients like myself may encounter when seeking treatment from mental health services again. The responsibility for repairing the harms of iatrogenic trauma is a shared one. Patients are doing their part by being willing to show up at all in settings that have previously harmed them. It is time that mental healthcare providers played their part alongside these patients. For this to be achieved, services need to be designed and resourced to facilitate clinicians to work confidently and compassionately with iatrogenic trauma rather than avoiding it.

This subject, after all, is a shared one – in more ways than we might think. The harms sustained by patients are enacted upon them, by or from something else, within a system. The mental healthcare system often inflicts harm resulting in trauma because it is itself a traumatised system, with environments that often harm staff as well as patients. Patients presenting with and naming the traumatic consequences of this system may make staff (who may also be harmed) feel deeply uncomfortable. Such staff may not feel similarly able to name the harmful nature of their service and so may deny this of patients too. Patients are not to blame for the problems that are experienced by them but not caused by them. To be compassionate in caring is to be alongside patients in their suffering,^32^ and accepting reality as it is may help create a greater sense of compassion for one another through recognising that this suffering is often shared. Rather than setting the needs of one group against the other, or being beset by fear of blame, we can aim for meeting one another as we are and relationships of mutual healing between clinicians and patients. When the wholeness and healing of each of us matters, the system may begin to heal too.

## About the author

**James Downs** is a patient representative at the Royal College of Psychiatrists, Cardiff, Wales, UK.
